# Non-Completely Displaced Traumatic Rib Fractures: Potentially Less Crucial for Pulmonary Adverse Outcomes, Regardless of Classification

**DOI:** 10.3390/medicina61010081

**Published:** 2025-01-06

**Authors:** Hongrye Kim, Su Young Yoon, Jonghee Han, Junepill Seok, Wu Seong Kang

**Affiliations:** 1 Department of Neurosurgery, Chungbuk National University Hospital, Cheongju 28644, Republic of Korea; nshrkim@gmail.com; 2 Department of Thoracic and Cardiovascular Surgery, Chungbuk National University Hospital, Cheongju 28644, Republic of Korea; ysy1227@hanmail.net (S.Y.Y.); medihan7@naver.com (J.H.); 3 Department of Thoracic and Cardiovascular Surgery, Chungbuk National University, College of Medicine, Cheongju 28644, Republic of Korea; 4 Department of Trauma Surgery, Jeju Regional Trauma Center, Cheju Halla General Hospital, Jeju 63127, Republic of Korea

**Keywords:** rib fracture, trauma, displacement, classification, criteria

## Abstract

*Background and Objectives*: Two major classification systems exist for rib fracture (RFX) displacement. One system uses a 50% displacement threshold: Grade I (<50%), Grade II (≥50% to <100%), and Grade III (completely dislocated). Another proposes a 10% threshold: Undisplaced (<10%), Offset (≥10% to <100%), and Displaced (completely dislocated). We analyzed risk factors for adverse outcomes for pulmonary complications and mortality according to both classification criteria. *Materials and Methods*: We retrospectively reviewed trauma registry and medical records from January 2019 to December 2023. All radiographic parameters were recorded based on initial computed tomography. Primary outcomes were pneumonia and other pulmonary complications requiring surgery. Least absolute shrinkage and selection operator (LASSO) regression was conducted to select risk factors and minimize overfitting. Multivariable logistic regression (MLR) was performed after LASSO. *Results*: Among the 621 patients, 61 (9.8%) had one or more adverse outcomes. In MLR, regardless of both classifications, the age (*p* < 0.001), ISS (*p* < 0.001), and number of completely displaced RFX (*p* = 0.001) were statistically significant. After excluding 280 patients with completely displaced RFX, we conducted a subgroup analysis with the remaining 341 patients. In this analysis, 22 (6.5%) patients experienced one or more adverse outcomes. Regardless of both classifications, the AIS head (*p* = 0.006), AIS extremities (*p* = 0.012), and number of segmental RFX (*p* < 0.001) were statistically significant in MLR. The area under the receiver operating curve for both MLR models was 0.757 in the total patient group and 0.823 in the subgroup that excluded patients with completely displaced RFX. *Conclusions*: Completely displaced RFX is the most crucial factor, regardless of the classification criteria. Unless ribs are completely displaced, the degree of displacement may not be crucial, and the number of segmental RFX was a significant risk factor.

## 1. Introduction

Rib fracture (RFX) is one of the most common injuries observed in patients with blunt chest trauma, found in approximately 40% of cases [1,2,3]. RFX is a well-known risk factor for various pulmonary complications as well as mortality [3,4,5]. Although likely confounded by other concurrent traumatic injuries, the mortality rate for RFX is substantial, approximately 10% for all ages [6,7]. In older patients, each additional RFX increases the risk of pneumonia by 27% and mortality by 19% compared to younger patients [8].

To date, in addition to the number of RFXs, factors such as the degree of displacement, associated pulmonary contusion (PC), and the presence of a flail segment have been studied as risk factors for adverse outcomes [4,5,9,10]. However, a clear standard for the degree of displacement in RFX has not yet been established.

Discussions regarding the terminology of RFX patterns are ongoing, and currently, there are two major discussions or bases for classifying RFX displacements. First, Chien et al. and other studies have suggested a classification that fractures are divided into Grade I and Grade II based on a “50% displacement threshold” (Grade I: RFX with a displacement of <50% of rib the width on axial computed tomography (CT); Grade II: between ≥50% and <100%), while fractures that are completely dislocated are classified as Grade III [4,11,12]. Second, Edwards et al. suggested another classification that fractures are divided into “Undisplaced” and “Offset” based on a “10% displacement threshold” (Undisplaced: RFX with a displacement of <10% of the rib width on axial CT; Offset: between ≥10% and <100%), while fractures that are completely dislocated are classified as “Displaced” [13].

Although both classification systems agree that completely displaced RFX is the most severe form of RFX, they differ significantly in their definition of partially displaced RFX. To date, no study has conclusively determined which classification system is superior.

In this study, we applied both the 50% and 10% threshold criteria to our study population and sought to have this classification reflect patient severity. We hypothesized that the 50% and 10% threshold criteria would exhibit a significant difference in sensitivity and specificity for predicting adverse outcomes. Additionally, we analyzed whether risk factors for adverse outcomes varied according to each classification criterion.

## 2. Materials and Methods

### 2.1. Study Design and Data Source

We retrospectively reviewed the trauma registry and medical records between January 2019 and December 2023 at the Chungbuk National University Hospital, Cheongju, South Korea. Our institution is a tertiary care, university-affiliated hospital with 930 beds, making it one of South Korea’s largest trauma centers. The hospital is responsible for 2.5 million people, with almost 450 patients presenting with an Injury Severity Score (ISS) > 15 annually [14]. This study was approved by the Institutional Review Board of Chungbuk National University Hospital (CBNUH 2024-11-011). All methods were performed following relevant guidelines and regulations. Patient information was analyzed anonymously; therefore, the requirement for informed consent was waived. We recorded data for all patients presenting with blunt chest trauma at the time of admission, including the ISS and Abbreviated Injury Scale (AIS) [15]. Patient progression, including pneumonia onset during the index hospitalization, was prospectively recorded. In our trauma center, we routinely performed CT scans from the head to the pelvis on all patients. RFX patterns and the degree of PC were recorded once based on initial chest CT performed by a thoracic surgeon affiliated with the trauma center with more than 10 years of experience. CT was also performed on the extremities if required. All patient data were encoded to ensure the participants’ privacy and data confidentiality.

### 2.2. Study Population and Inclusion and Exclusion Criteria

**Figure 1:**
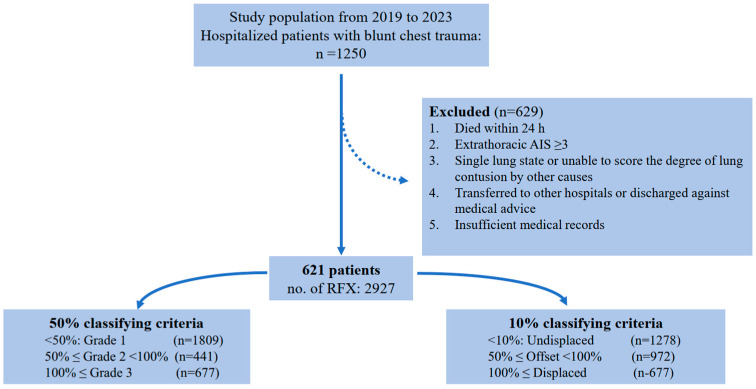
Flowchart for patient selection.

This study enrolled consecutive patients with blunt chest trauma who presented to our trauma center during the study period. Patients who did not survive >24 h were excluded. Additional exclusion criteria included (a) patients with extrathoracic injuries with AIS ≥ 3; (b) conditions where the degree of PC could not be assessed, such as a collapsed lung due to tension pneumothorax or one lung state due to a previous history of pneumonectomy; (c) discharge against medical advice; and (d) insufficient medical records (Figure 1).

### 2.3. Definitions

The RFX location was divided into three parts using the anterior and posterior axillary lines [13]. Segmental RFX was diagnosed when a single rib had ≥2 fractures at different locations. Flail chest was subclassified and defined as follows: (a) anatomical flail segment: radiologically confirmed three or more consecutive segmental RFX; (b) flail motion: clinically confirmed paradoxical movement of the chest wall during the index hospitalization.

The degree of PC was scored using the blunt pulmonary contusion score (BPC18), [10,16], which divides each lung field into upper, middle, and lower thirds. Each third received a score of 0–3, based on the density of the affected lung (Supplementary Figure S1).

As mentioned above, the degree of RFX displacement was determined using both the 50% threshold (Grades 1, 2, and 3) and 10% threshold (Undisplaced, Offset, and Displaced), each as a separate dataset (Supplementary Figure S2).

### 2.4. Primary Outcome

The primary outcome of our study was defined as one or more of the following pulmonary adverse outcomes: (a) pneumonia and (b) pulmonary complications requiring surgical treatment, such as empyema, injuries of the descending aorta or diaphragm due to RFX, or massive hemothorax.

### 2.5. Statistical Analysis

All statistical analyses were performed using R version 4.2 (R Foundation, Vienna, Austria) [17]. Means and averages were used to represent continuous data, whereas proportions were used to represent categorical data. Continuous data were compared using Student’s *t*-test or Mann–Whitney U test. Proportions were compared using the chi-square test or Fisher’s exact test, as appropriate. Statistical significance was set at *p* < 0.05. However, for some skewed data, such as the length of stay in the intensive care unit, the median and interquartile range (IQR) were applied.

In this study, we used the least absolute shrinkage and selection operator (LASSO) regression to minimize overfitting [18,19]. We performed a tenfold cross-validation to select an optimal hyperparameter (λ). In the cross-validation, optimal λ was selected as the most regularized model so that the error was within one standard error of the minimum. Risk factors that showed statistical significance (*p* < 0.05) in the univariate analyses of the primary outcome were entered into the LASSO regression model. After parameter selection using LASSO regression, we conducted multivariable logistic regression (MLR) using the selected features. We used a receiver operating characteristic (ROC) curve to investigate the performance of the prediction models and calculate the area under the ROC curve (AUROC).

## 3. Results

### 3.1. Patient Characteristics

The baseline characteristics and outcomes of the study population are shown in Table 1. During the study period, 1250 patients with chest AIS > 0 presented; of these, 621 were finally included in the analysis (Figure 1). Among them, 61 (9.8%) patients had one or more adverse outcomes and were classified as the primary outcome group. A total of 33 (5.3%) patients had pneumonia, and the same number of patients required surgery for pulmonary complications. The majority of patients were males (463, 74.6%). The average age was 58.9 ± 16.6 years. The average head and chest AIS scores were 0.3 ± 0.7 and 2.9 ± 0.6, respectively. These parameters showed a statistically significant difference for the adverse pulmonary outcomes along with the AIS extremities and ISS (73% vs. 88.5%, *p* = 0.013; 58.0 ± 16.5 vs. 66.6 ± 14.7, *p* < 0.001; 0.2 ± 0.6 vs. 0.5 ± 0.9, *p* = 0.011; 2.8 ± 0.6 vs. 3.1 ± 0.5, *p* < 0.001; 0.7 ± 0.9 vs. 1.0 ± 1.0, *p* < 0.031; 11.7 ± 4.2 vs. 14.1 ± 4.5, *p* < 0.001, respectively). Flail segments (39.3% vs. 22.9%, *p* = 0.007), flail motion (27.9% vs. 3.2%, *p* < 0.001), and hemothorax (68.9% vs. 46.2%, *p* = 0.001) were observed more frequently in patients with adverse pulmonary outcomes. The body mass index, injured side of the chest wall, and presence of pneumothorax were not significantly different between the two groups.

### 3.2. Comparison Between Patients with and Without Pulmonary Adverse Outcomes

Table 2 presents the univariable analyses of the parameters that changed according to the application of the 50% threshold criteria (<50%: Grade 1; 50% ≤ Grade 2 < 100%; <100%: Grade 3) and 10% threshold criteria (<10%: ‘Undisplaced’; 10% ≤ ‘Offset’ < 100%; <100%: ‘Displaced’). Among the 621 patients, 2927 rib fractures were observed. According to the 50% threshold criterion, there were 1809 Grade 1 fractures, 441 Grade 2 fractures, and 677 Grade 3 fractures. Under the 10% threshold criterion, 531 Grade 1 fractures were upgraded by one grade, resulting in 1278 ‘Undisplaced’ fractures, 972 ‘Offset’ fractures, and 677 ‘Displaced’ fractures. Completely displaced RFX was evaluated as the highest grade under both criteria, resulting in the same number of Grade 3 and ‘Displaced’ fractures. However, fractures that changed grades according to the criteria did not show statistical significance in the primary outcome. Among RFX patterns, only the total number of RFXs (4.5 ± 3.0 vs. 6.4 ± 3.6, *p* < 0.001), the number of segmental fractures (1.3 ± 2.0 vs. 2.4 ± 2.5, *p* < 0.001), and the most severe grade fractures (Grade 3 and ‘Displaced’; 1.0 ± 1.5 vs. 2.1 ± 2.6, *p* < 0.001) showed statistical significance for the primary outcome.

### 3.3. Risk Factor Analysis by MLR After LASSO

LASSO identified the most important risk factors (Supplementary Figure S3). For both the 50% and 10% threshold criteria, only three parameters—age, ISS, and number of completely displaced RFX—were selected by LASSO regression. In multivariable logistic analysis, age (1.04 OR, 95% CI (1.02–1.06), *p* < 0.001), ISS (1.12 OR, 95% CI (1.05–1.20), *p* < 0.001), and number of completely displaced RFXs (1.25 OR, 95% CI (1.09–1.44), *p* = 0.001) were statistically significant. These three parameters did not change in value regardless of the threshold criteria, resulting in the same outcome from the LASSO regression for both the 50% and 10% threshold criteria. Consequently, among RFX patterns, regardless of whether the 50% or 10% threshold criteria were applied, only the number of completely displaced RFXs (Grade 3 and ‘Displaced’) showed statistical significance (Table 3).

### 3.4. Subgroup Analysis After Excluding Patients with Grade 3 (Or Displaced) Fracture

We conducted subgroup analysis by excluding patients with completely displaced RFXs. Baseline characteristics and comparison between patients with or without adverse pulmonary outcomes were summarized in Table 4. Univariable analysis for the primary outcome according to different RFX patterns of the 50% and 10% threshold criteria in patients without completely displaced rib fractures were summarized in Table 5. Of note, the number of RFXs and segmental RFXs were significant risk factors in both classifications. We also conducted LASSO logistic regression, which revealed that three parameters (AIS head, AIS extremities, and the number of segmental RFXs) were statistically significant for adverse pulmonary outcomes (Supplementary Figure S4). We then developed a logistic regression model based on these three parameters: AIS head (2.07 OR, 95% CI (1.23–3.49), *p* = 0.006), AIS extremities (1.85 OR, 95% CI (1.15–3.00), *p* = 0.012), and number of segmental RFXs (1.66 OR, 95% CI (1.25–2.21), *p* < 0.001, Table 6).

### 3.5. Model Performance

**Figure 2:**
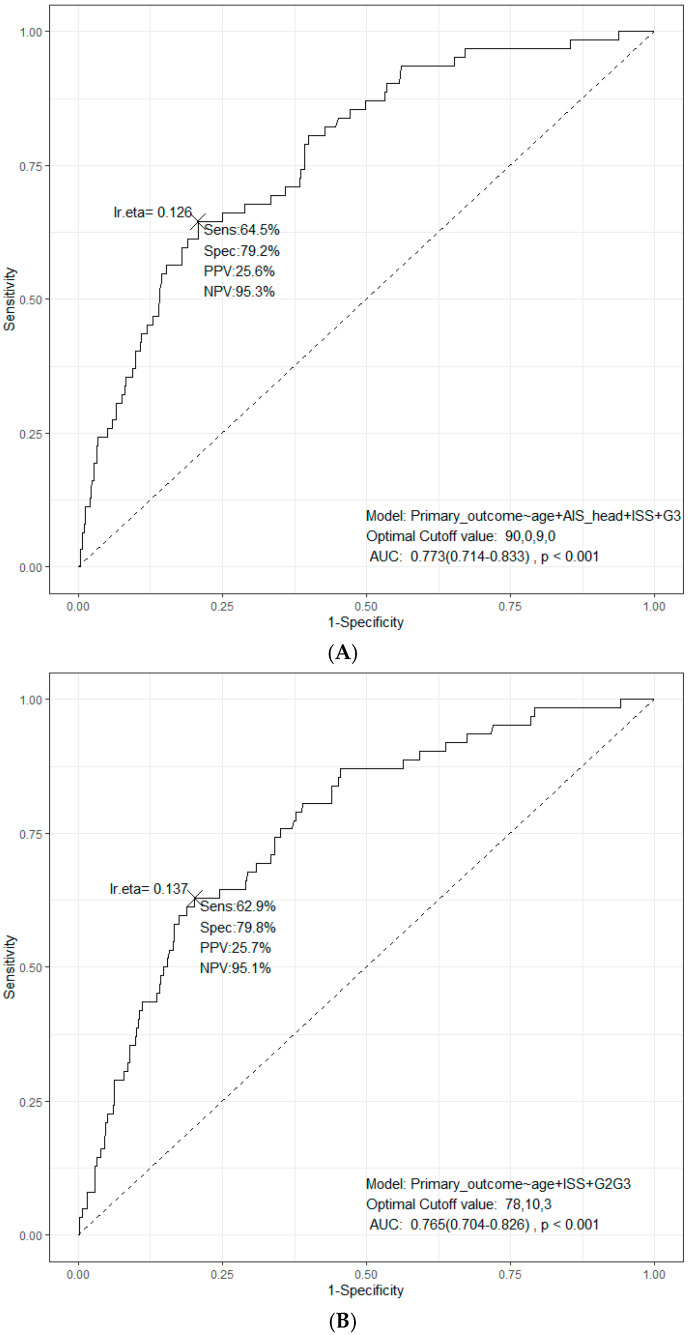

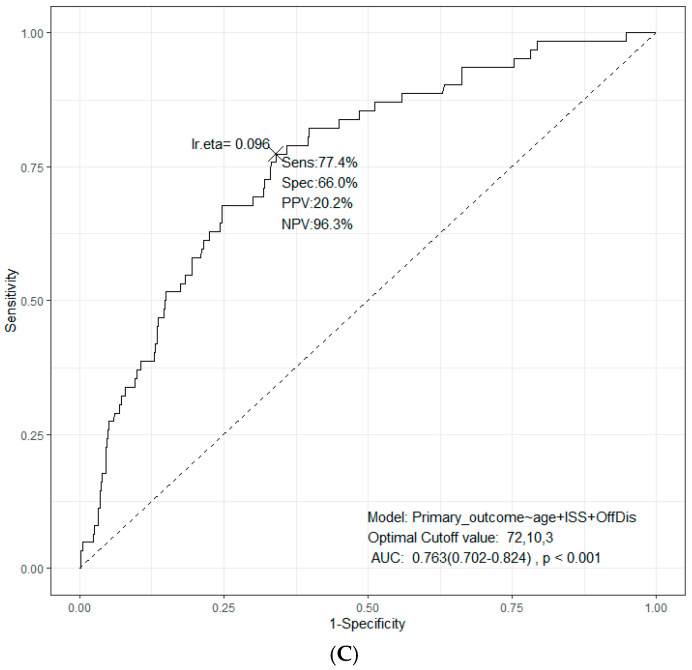
Receiver operating characteristic (ROC) analysis for the primary outcome. (**A**) ROC curve for the original parameters, including age, AIS head, ISS, and completely displaced RFXs. (**B**) ROC curve for the 50% threshold criteria with a combined parameter (G2 or higher), excluding the parameter of completely displaced RFXs. (**C**) ROC curve for the 10% threshold criteria with a combined parameter (Offset or higher), excluding the parameter of completely displaced RFXs.

The model performance of the multivariable logistic model is shown in Figure 2. Figure 2A shows the ROC curve of the model comprising all patients, with a sensitivity, specificity, and AUC of 64.5%, 79.2%, and 0.753, respectively. Figure 2B shows the ROC curve for the 50% threshold criteria with a combined parameter (G2 or higher), excluding the parameter of completely displaced RFXs, with a sensitivity, specificity, and AUC of 62.9%, 79.8%, and 0.765, respectively. Figure 2C shows the ROC curve for the 10% threshold criteria with a combined parameter (Offset or higher), excluding the parameter of completely displaced RFXs, with a sensitivity, specificity, and AUC of 77.4%, 66.0%, and 0.763, respectively.

## 4. Discussion

To the best of our knowledge, this is the first study to analyze the classification criteria for the degree of displacement of RFXs. A previous study indicated that the distinction between the 50% and 10% threshold criteria for the occurrence of flail motion was not significant [12]. Herein, we found that, regardless of whether the 50% or 10% threshold criteria were used, only completely displaced RFX was statistically significant for both classification criteria among the RFX patterns, similarly to the results of a previous study. Of note, in our subgroup analysis, the number of segmental RFXs was a significant risk factor, whereas the distinction between the 50% and 10% threshold of displacement was not significant. Our study suggests that, in patients without completely displaced or segmental rib fractures (RFXs), the number of RFXs and the degree of displacement have no clinical significance. We believe this finding will aid in future decisions regarding hospitalization and treatment. However, a larger prospective study is warranted regarding this issue.

Many studies have reported that the number of RFXs, with or without displacement, is closely associated with adverse outcomes [20,21,22]. However, some studies suggest that the number of rib fractures is not a significant risk factor [3,7]. Similarly, in our study, the total number of RFXs was not a risk factor for adverse pulmonary outcomes, and only completely displaced fractures among the detailed fracture patterns were statistically significant. However, we believe that the total number of RFXs as a parameter can sometimes be statistically significant, depending on the context. Considering that as the number of broken ribs increases, the likelihood of having more severely broken ribs also increases. Among the various RFX patterns, simply analyzing based on the number of RFXs may show statistical significance. However, if a stronger parameter than the number of RFXs is present in the RFX patterns and is included in the analysis, the number of RFXs may lose its statistical significance. Therefore, efforts to identify the most significant risk factors of various RFX patterns should continue [5,12].

In 2017, Chien et al. reported that an RFX displacement of >50% was statistically significant for adverse outcomes [4], and subsequent studies have also been reported based on the 50% threshold criteria [5,11,12]. In 2020, Edwards et al. [13] proposed the 10% threshold criteria, which have since been externally validated. In this study, we aimed to compare the accuracy of prediction models for adverse pulmonary outcomes when the displacement grading criteria changed from the 50% to 10% threshold. As a result, neither the Grade 1 (<50%) or Grade 2 (50% ≤ x < 100%) nor ‘Undisplaced’ (<10%) or ‘Offset’ (10% ≤ x < 100%) diagnostic criteria showed significant associations with adverse outcomes. Given that our study found no statistical significance between non-completely displaced RFXs and adverse pulmonary outcomes, classifying RFX displacement based on the 10% or 50% threshold criteria may be meaningless. However, our study is limited in short-term outcomes; therefore, a long-term follow-up study is warranted.

Of note, the number of segmental RFXs was a significant risk factor even in low-graded RFXs. Generally, segmental and comminuted fractures would occur when a bone is hit at two points or by a large surface, or would result from relatively high levels of force [23]. From this perspective, even patients with only low-grade RFX (less than 100% displacement) may have sustained a broader area and greater impact if they have a higher number of segmental RFXs compared to others. Whether a greater amount of impact is required to cause completely dislocated RFX or segmental RFX remains unstudied. A previous study analyzing the risk factors for flail motion of the chest wall reported that the number of segmental RFXs composed of completely dislocated RFX is significant [12]. Further studies are needed to determine the most appropriate diagnostic criteria for clinical practice.

Our study had several limitations. First, the retrospective design may have induced selection bias because the excluded patients who died within 24 h may have had severe rib fractures classified as the most severe form. Second, RFX patterns were recorded once based on the initial chest CT. As the degree of RFX displacement changes over time, follow-up with repeat chest CT is necessary; however, we could not perform chest CT scans because of cost and patient safety. [24] Third, this was a single-cohort study, and external validation was not conducted. Fourth, the severity of pneumonia as the primary outcome may vary. However, in our cohort, all patients with pneumonia received antibiotic treatment and exhibited a higher likelihood of longer hospital and ICU stays, and prolonged mechanical ventilation. We hypothesized that every type of pneumonia represents adverse outcomes and could potentially serve as a precursor to ARDS or multi-organ failure. In future studies, a stratified analysis is warranted. Fifth, we evaluated the severity of RFX using pulmonary adverse outcomes. However, the degree of injury severity may be evaluated with various outcomes such as the length of hospital stay, pain score, and the amounts of analgesics during index hospitalization, or mortality. Sixth, our statistical analysis did not prove the difference among non-completely displaced RFX. Further larger scale study is warranted to identify the exact effect size of this RFX patten. Seventh, to conduct this study with patients as close as possible to isolated chest trauma cases, we excluded patients with severe extra-thoracic injuries (AIS ≥ 3). However, the ISS was still a significant risk factor for pulmonary complications in the overall group. In the subgroup analysis, extremity AIS was identified as a significant risk factor. These findings suggest that in patient groups requiring prolonged bed rest, such as those with pelvic or long bone fractures, immobility may have contributed to the development of pneumonia as a complication. In future studies, we plan to explore a more comprehensive model that considers severe brain and abdominal injuries. Finally, we did not conduct a subgroup analysis based on comorbidities, the occurrence of osteoporosis, follow-up period, or T-score values. Moreover, pulmonary complications related to these conditions were not investigated. Future studies on this issue are needed.

## 5. Conclusions

Our study demonstrated that “completely displaced” RFX is the most important factor, regardless of the classification criteria. Unless ribs were completely displaced, the degree of displacement may not be crucial. Of note, the number of segmental RFX was a significant risk factor in patients without completely displaced RFX, whereas displacement of RFX < 100% was not significant. Further study is required to determine the severity of low-graded RFX.

## Figures and Tables

**Table 1:** Univariate analysis of baseline characteristics for the primary outcome: common parameters for the 50% threshold criteria and 10% threshold criteria.

		Total Patients (n = 621)	No Adverse Outcome (n = 560, 90.2%)	Adverse Outcome (n = 61, 9.8%)	*p*
sex	F	158 (25.4%)	151 (27%)	7 (11.5%)	0.013
	M	463 (74.6%)	409 (73%)	54 (88.5%)	
age	Mean ± SD	58.9 ± 16.6	58.0 ± 16.5	66.6 ± 14.7	<0.001
BMI	Mean ± SD	24.1 ± 3.9	24.1 ± 3.8	24.1 ± 4.4	0.954
AIS head	Mean ± SD	0.3 ± 0.7	0.2 ± 0.6	0.5 ± 0.9	0.011
AIS face	Mean ± SD	0.2 ± 0.5	0.2 ± 0.5	0.2 ± 0.6	0.346
AIS chest	Mean ± SD	2.9 ± 0.6	2.8 ± 0.6	3.1 ± 0.5	<0.001
AIS abdomen	Mean ± SD	0.4 ± 0.8	0.4 ± 0.8	0.3 ± 0.7	0.293
AIS extremities	Mean ± SD	0.7 ± 0.9	0.7 ± 0.9	1.0 ± 1.0	0.031
AIS external	Mean ± SD	0.5 ± 0.5	0.5 ± 0.5	0.6 ± 0.6	0.215
ISS	Mean ± SD	11.9 ± 4.3	11.7 ± 4.2	14.1 ± 4.5	<0.001
BPC18	Mean ± SD	1.6 ± 2.5	1.5 ± 2.3	2.5 ± 3.9	0.060
Side	Left	307 (49.4%)	277 (49.5%)	30 (49.2%)	1.000
	Right	314 (50.6%)	283 (50.5%)	31 (50.8%)	
Flail segment		152 (24.5%)	128 (22.9%)	24 (39.3%)	0.007
Flail motion		35 (5.6%)	18 (3.2%)	17 (27.9%)	<0.001
Pneumothorax		298 (48.0%)	266 (47.5%)	32 (52.5%)	0.548
Hemothorax		301 (48.5%)	259 (46.2%)	42 (68.9%)	0.001
LOS hospital, day	Median [IQR]	9.0 [5.1–16.8]	8.8 [5.0–15.0]	21.0 [16.0–33.0]	<0.001
LOS ICU, hr	Median [IQR]	0.0 [0.0–37.2]	0.0 [0.0–25.1]	77.8 [0.0–225.8]	<0.001
LOS MV, hr	Median [IQR]	0.0 [0.0–0.0]	0.0 [0.0–0.0]	0.0 [0.0–60.8]	<0.001
Overall adverse outcome		61 (9.8%)			
Pneumonia		33 (5.3%)			
Requiring surgery		33 (5.3%)			

n: number; SD: standard deviation; BMI: body mass index; IQR: interquartile ranges; BPC18: blunt pulmonary contusion score; ICU: intensive care unit; MV: mechanical ventilator; LOS: length of stay; ISS: Injury Severity Score; AIS: Abbreviated Injury Scale.

**Table 2:** Univariate analysis of baseline characteristics for the primary outcome: different rib fracture patterns of the 50% threshold criteria and 10% threshold criteria.

50% Threshold (<50%: Grade 1; 50% < Grade 2 < 100%; <100%: Grade 3)					
		Total Patients (n = 621)	No Adverse Outcome (n = 560)	Adverse Outcome (n = 61)	*p*
No. of RFXs (n = 2927)	Mean ± SD	4.7 ± 3.1	4.5 ± 3.0	6.4 ± 3.6	<0.001
Grade 1 (n = 1809)	Mean ± SD	2.9 ± 2.3	2.9 ± 2.3	3.3 ± 2.8	0.223
Grade 2 (n = 441)	Mean ± SD	0.7 ± 1.1	0.7 ± 1.0	0.9 ± 1.1	0.172
Grade 3 (n = 677)	Mean ± SD	1.1 ± 1.7	1.0 ± 1.5	2.1 ± 2.6	<0.001
No. of segmental RFXs (n = 799)	Mean ± SD	1.3 ± 2.0	1.2 ± 1.9	2.4 ± 2.5	<0.001
**10% Threshold** **(<10%: Undisplaced; 10% < Offset < 100%; <100%: Displaced)**					
		**Total Patients (n = 621)**	**No Adverse Outcome** **(n = 560)**	**Adverse Outcome** **(n = 61)**	***p***
No. of RFXs (n = 2927)	Mean ± SD	4.7 ± 3.1	4.5 ± 3.0	6.4 ± 3.6	<0.001
Undisplaced (n = 1278)	Mean ± SD	2.1 ± 2.0	2.0 ± 1.9	2.3 ± 2.3	0.356
Offset (n = 972)	Mean ± SD	1.6 ± 1.6	1.5 ± 1.6	1.9 ± 1.8	0.058
Displaced (n = 677)	Mean ± SD	1.1 ± 1.7	1.0 ± 1.5	2.1 ± 2.6	<0.001
No. of segmental RFXs (n = 799)	Mean ± SD	1.3 ± 2.0	1.2 ± 1.9	2.4 ± 2.5	<0.001

n and No.: number; SD: standard deviation; RFX: rib fracture.

**Table 3:** Multiple logistic regression model using three parameters that were selected in LASSO regression.

	Univariable			Multivariable		
	OR	95% CI	*p*	OR	95% CI	*p*
age	1.04	(1.02–1.06)	<0.001	1.04	(1.02–1.06)	<0.001
ISS	1.13	(1.07–1.20)	<0.001	1.12	(1.05–1.20)	<0.001
No. of completely displaced RFXs	1.35	(1.19–1.54)	<0.001	1.25	(1.09–1.44)	0.001

LASSO: least absolute shrinkage and selection operator; No.: number; OR: odds ratio; CI: confidence interval; ISS: Injury Severity Score; RFX: rib fracture.

**Table 4:** Univariate analysis of baseline characteristics for the primary outcome: common parameters for the 50% and 10% threshold criteria in patients without completely displaced rib fractures.

		Patients Without Totally Displaced RFXs (n = 341)	No Adverse Outcome (n = 319, 93.5%)	Adverse Outcome (n = 22, 6.5%)	*p*
sex	F	89 (26.1%)	88 (27.6%)	1 (4.5%)	0.033
	M	252 (73.9%)	231 (72.4%)	21 (95.5%)	
age	Mean ± SD	57.4 ± 17.7	56.8 ± 17.6	65.2 ± 17.1	0.031
BMI	Mean ± SD	24.3 ± 3.8	24.2 ± 3.8	25.7 ± 4.0	0.076
AIS head	Mean ± SD	0.3 ± 0.7	0.2 ± 0.6	0.7 ± 1.0	0.030
AIS face	Mean ± SD	0.2 ± 0.6	0.2 ± 0.6	0.1 ± 0.4	0.361
AIS chest	Mean ± SD	2.7 ± 0.7	2.7 ± 0.7	3.0 ± 0.4	0.007
AIS abdomen	Mean ± SD	0.5 ± 0.8	0.5 ± 0.8	0.4 ± 0.8	0.578
AIS extremities	Mean ± SD	0.6 ± 0.9	0.5 ± 0.9	1.3 ± 1.0	<0.001
AIS external	Mean ± SD	0.5 ± 0.5	0.5 ± 0.5	0.6 ± 0.5	0.307
ISS	Mean ± SD	11.2 ± 4.4	11.0 ± 4.3	14.3 ± 3.6	<0.001
BPC18	Mean ± SD	1.3 ± 2.3	1.3 ± 2.3	1.5 ± 3.2	0.738
Side	L	160 (46.9%)	146 (45.8%)	14 (63.6%)	0.160
	R	181 (53.1%)	173 (54.2%)	8 (36.4%)	
Flail segment		29 (8.5%)	23 (7.2%)	6 (27.3%)	0.004
Flail motion		4 (1.2%)	1 (0.3%)	3 (13.6%)	<0.001
Pneumothorax		129 (37.8%)	120 (37.6%)	9 (40.9%)	0.936
Hemothorax		107 (31.4%)	95 (29.8%)	12 (54.5%)	0.029
LOS hospital, day	Median [IQR]	7.0 [4.1–14.0]	7.0 [4.0–13.0]	20.8 [16.0–34.0]	<0.001
LOS ICU, hr	Median [IQR]	0.0 [0.0–24.8]	0.0 [0.0–19.1]	125.0 [35.8–353.2]	<0.001
LOS MV, hr	Median [IQR]	0.0 [0.0–0.0]	0.0 [0.0–0.0]	0.0 [0.0–47.5]	<0.001
Overall adverse outcome		22 (6.5%)			
Pneumonia		12 (3.5%)			
Requiring surgery		11 (3.2%)			

n: number; SD: standard deviation; BMI: body mass index; IQR: interquartile ranges; BPC18: blunt pulmonary contusion score; ICU: intensive care unit; MV: mechanical ventilator; LOS: length of stay; RFX: rib fracture; ISS: Injury Severity Score; AIS: Abbreviated Injury Scale.

**Table 5:** Univariate analysis of baseline characteristics for the primary outcome: different rib fracture patterns of the 50% and 10% threshold criteria in patients without completely displaced rib fractures.

50% Threshold (<50%: Grade 1; 50% < Grade 2 < 100%; >100%: Grade 3)					
		Total Patients (n = 341)	No Adverse Outcome (n = 319)	Adverse Outcome (n = 22)	*p*
No. of RFXs (n = 1245)	Mean ± SD	3.7 ± 2.5	3.5 ± 2.4	5.3 ± 3.6	0.034
Grade 1 (n = 1064)	Mean ± SD	3.1 ± 2.3	3.0 ± 2.2	4.7 ± 3.4	0.032
Grade 2 (n = 181)	Mean ± SD	0.5 ± 0.9	0.5 ± 0.9	0.6 ± 0.9	0.586
Patients with two or more Grade 2 RFXs	n (%)	43 (12.6%)	39 (12.2%)	4 (18.2%)	0.630
Patients with three or more Grade 2 RFXs	n (%)	15 (4.4%)	14 (4.4%)	1 (4.5%)	1.000
No. of segmental RFXs (n = 165)	Mean ± SD	0.5 ± 1.1	0.4 ± 1.0	1.7 ± 2.1	0.011
**10% Threshold** **(<10%: Undisplaced; 10% < Offset < 100%;** **>100%: Displaced)**					
		**Total Patients** **(n = 341)**	**No Adverse Outcome** **(n = 319)**	**Adverse Outcome** **(n = 22)**	***p***
No. of RFXs (n = 1245)	Mean ± SD	3.7 ± 2.5	3.5 ± 2.4	5.3 ± 3.6	0.034
Undisplaced (n = 798)	Mean ± SD	2.3 ± 2.1	2.3 ± 2.0	3.4 ± 3.0	0.097
Offset (n = 447)	Mean ± SD	1.3 ± 1.5	1.3 ± 1.4	1.9 ± 1.6	0.046
Patients with two or more Offset RFXs	n (%)	127 (37.2%)	114 (35.7%)	13 (59.1%)	0.050
Patients with three or more Offset RFXs	n (%)	69 (20.2%)	61 (19.1%)	8 (36.4%)	0.094
No. of segmental RFXs (n = 165)	Mean ± SD	1.3 ± 2.0	0.4 ± 1.0	1.7 ± 2.1	0.011

n and No.: number; SD: standard deviation; RFX: rib fracture.

**Table 6:** Multiple logistic regression model using parameters that were selected in LASSO regression, in patients without completely displaced rib fractures.

	Univariable			Multivariable		
	OR	95% CI	*p*	OR	95% CI	*p*
AIS head	2.10	(1.31–3.36)	0.002	2.07	(1.23–3.49)	0.006
AIS extremities	2.20	(1.40–3.47)	<0.001	1.85	(1.15–3.00)	0.012
No. of segmental RFXs	1.74	(1.34–2.25)	<0.001	1.66	(1.25–2.21)	<0.001

LASSO: least absolute shrinkage and selection operator; No.: number; OR: odds ratio; CI: confidence interval; AIS: Abbreviated Injury Scale; RFX: rib fracture.

## Data Availability

Data are contained within the article and supplementary materials.

## Supplementary Materials

The following supporting information can be downloaded at: https://www.mdpi.com/article/10.3390/medicina61010081/s1, Supplementary Figure S1. Example of blunt pulmonary contusion scores. (A) A focal lung parenchymal contusion with a small pneumatocele in the left upper lobe (LUL) was identified. The LUL was assigned a blunt pulmonary contusion (BPC) score of 1. (B) Diffuse, multifocal lung parenchymal contusions in the right middle lobe (RML). The RML was assigned a BPC score of 2. (C) Diffuse haziness and a pneumatocele are observed throughout the entire right lung. Especially, the right lower lung of this patient was assigned a BPC score of 3. Supplementary Figure S2. Example of the degree of rib fracture displacement. (A) Initial three-dimensional chest computed tomography (3D chest CT) of a patient on admission. (B) On the axial cut of the chest CT, a slight displacement of the sixth rib is observed. The rib measures 9.4 mm in thickness, and the displacement is measured at 1.4 mm, corresponding to approximately 15% displacement. (C) On another axial cut, the seventh rib is completely displaced (100%). Supplementary Figure S3. Parameters were selected using LASSO logistic regression model. (A) Shrinkage of coefficients by hyperparameter (λ) using 50% threshold. (B) Hyperparameter selection (λ) using cross-validation using 50% threshold. The dotted line indicates the value of the harmonic log (λ) when the model error is minimized. In the LASSO logistic regression model using 50% threshold, three parameters were selected when log(λ) was −3.1454. (C) Shrinkage of coefficients by hyperparameter (λ) using 90% threshold. (D) Hyperparameter selection (λ) using cross-validation using 90% threshold. The dotted line indicates the value of the harmonic log (λ) when the model error is minimized. The same three parameters were also selected In the LASSO logistic regression model using 90% threshold, when log(λ) was −3.1454. Supplementary Figure S4. Parameters were selected using LASSO logistic regression model in subgroup analysis excluding patients with completely displaced RFX. (A) Shrinkage of coefficients by hyperparameter (λ) using 50% threshold. (B) Hyperparameter selection (λ) using cross-validation using 50% threshold. The dotted line indicates the value of the harmonic log (λ) when the model error is minimized. In the LASSO logistic regression model using 50% threshold, three parameters were selected when log(λ) was −3.249. (C) Shrinkage of coefficients by hyperparameter (λ) using 90% threshold. (D) Hyperparameter selection (λ) using cross-validation using 90% threshold. The dotted line indicates the value of the harmonic log (λ) when the model error is minimized. The same three parameters were also selected In the LASSO logistic regression model using 90% threshold, when log(λ) was −3.435.
