# Supplementary material for: Non-Completely Displaced Traumatic Rib Fractures: Potentially Less Crucial for Pulmonary Adverse Outcomes, Regardless of Classification
Source: Medicina (Kaunas). 2025 Jan 6;61(1):81. doi: 10.3390/medicina61010081 (PMC11767142; doi:10.3390/medicina61010081)
Supplement: Supplementary file 1 [file medicina-61-00081-s001.zip › medicina-3401002-supplementary.pdf]

Supplementary Figure S1. Example of blunt pulmonary contusion scores . (A) A focal lung parenchymal contusion with a small pneumatocele in the left upper lobe (LUL) was identified. The LUL was assigned a blunt pulmonary contusion (BPC) score of 1. (B) Diffuse, multifocal lung parenchymal contusions in the right middle lobe (RML). The RML was assigned a BPC score of 2. (C) Diffuse haziness and a pneumatocele are observed throughout the entire right lung. Especially, the right lower lung of this patient was assigned a BPC score of 3.

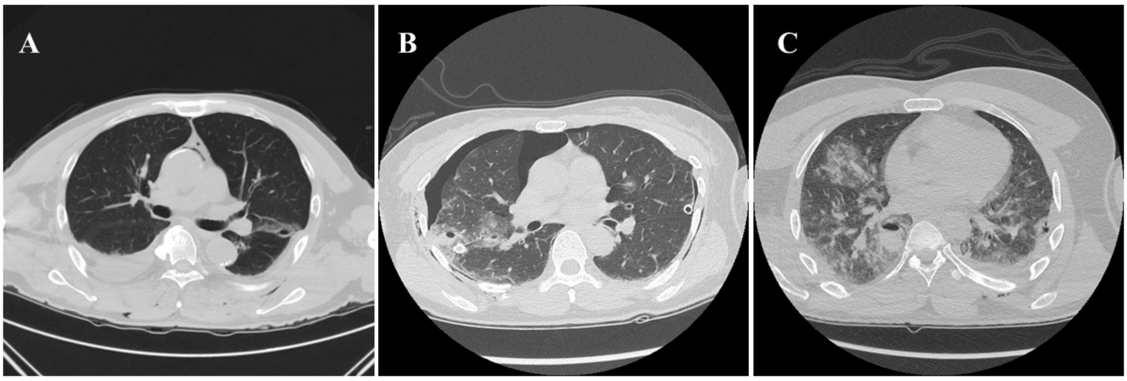

Supplementary Figure S2. Example of the degree of rib fracture displacement. (A) Initial three-dimensional chest computed tomography (3D chest CT) of a patient on admission. (B) On the axial cut of the chest CT, a slight displacement of the sixth rib is observed. The rib measures 9.4 mm in thickness, and the displacement is measured at 1.4 mm, corresponding to approximately 15% displacement. (C) On another axial cut, the seventh rib is completely displaced (100%).

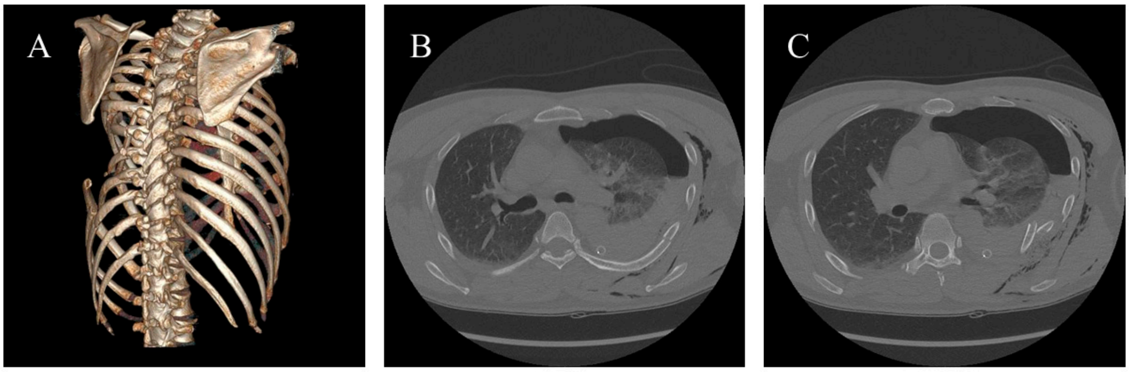

Supplementary Figure S3. Parameters were selected using LASSO logistic regression model. (A) Shrinkage of coefficients by hyperparameter (λ) using 50% threshold. (B) Hyperparameter selection (λ) using cross-validation using 50% threshold. The dotted line indicates the value of the harmonic log (λ) when the model error is minimized. In the LASSO logistic regression model using 50% threshold, three parameters were selected when log(λ) was -3.1454. (C) Shrinkage of coefficients by hyperparameter (λ) using 90% threshold. (D) Hyperparameter selection (λ) using cross-validation using 90% threshold. The dotted line indicates the value of the harmonic log (λ) when the model error is minimized. The same three parameters were also selected In the LASSO logistic regression model using 90% threshold, when log(λ) was -3.1454.

(A)

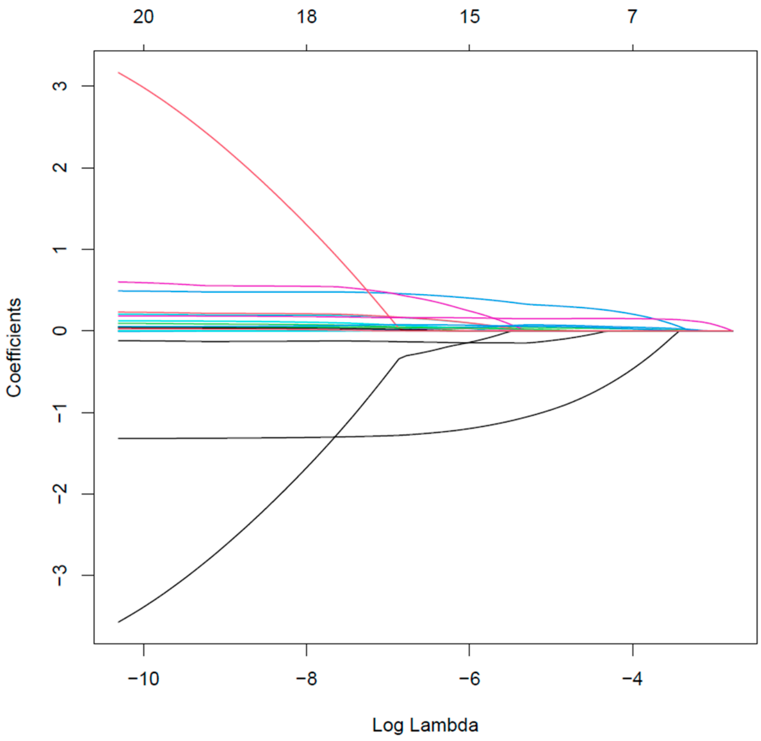
20
18
15
7
Coefficients
Log Lambda
−10
−8
−6
−4
3
2
1
0
−1
−2
−3

(B)

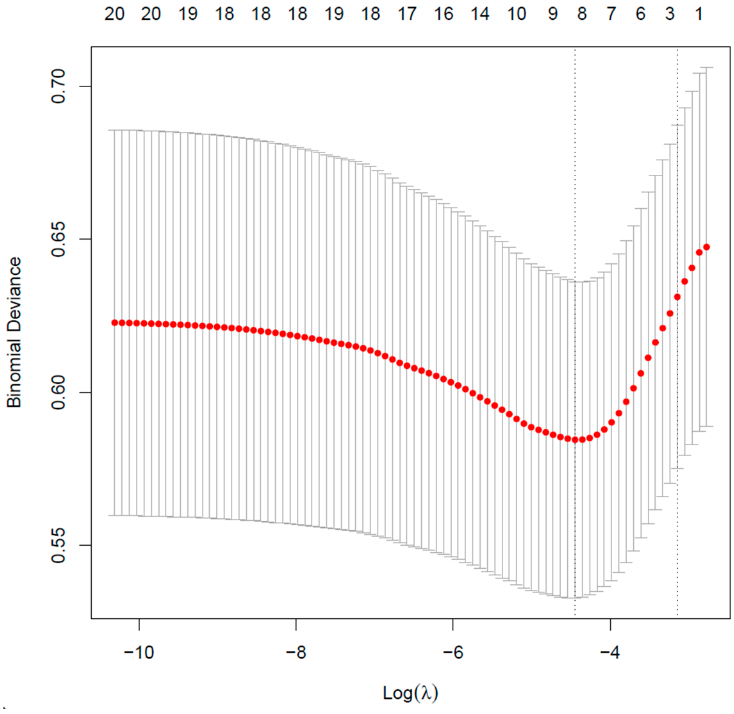
20 20 19 18 18 18 19 18 17 16 14 10 9 8 7 6 3 1
Binomial Deviance
0.70
0.65
0.60
0.55
−10
−8
−6
−4
Log(λ)

(C)

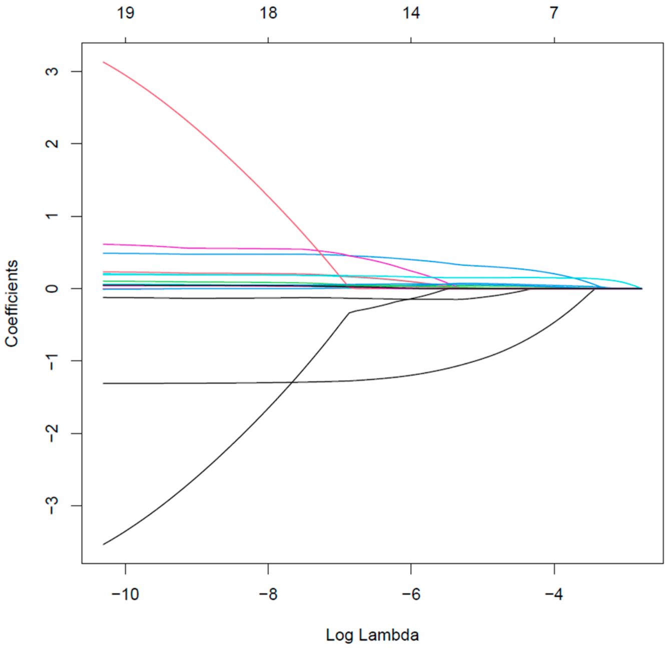

(D)

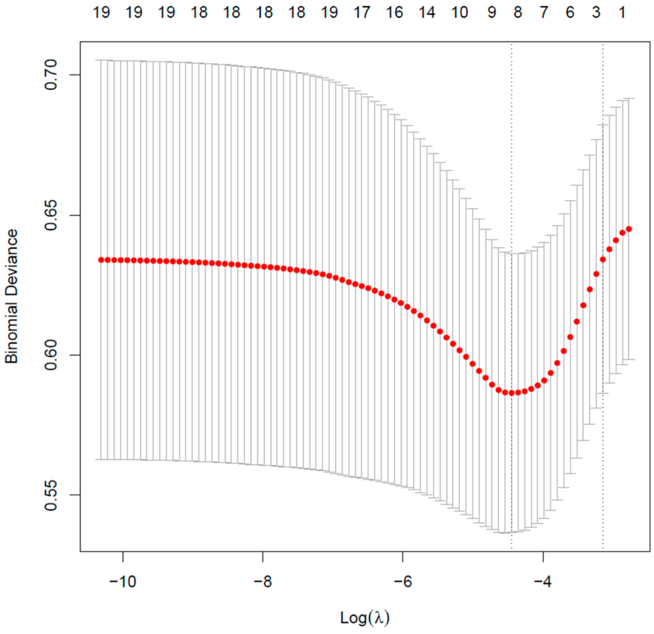

Supplementary Figure S4. Parameters were selected using LASSO logistic regression model in subgroup analysis excluding patients with completely displaced RFX. (A) Shrinkage of coefficients by

hyperparameter (λ) using 50% threshold. (B) Hyperparameter selection (λ) using cross-validation using 50% threshold. The dotted line indicates the value of the harmonic log (λ) when the model error is minimized. In the LASSO logistic regression model using 50% threshold, three parameters were selected when log(λ) was -3.249. (C) Shrinkage of coefficients by hyperparameter (λ) using 90% threshold. (D) Hyperparameter selection (λ) using cross-validation using 90% threshold. The dotted line indicates the value of the harmonic log (λ) when the model error is minimized. The same three parameters were also selected In the LASSO logistic regression model using 90% threshold, when log(λ) was -3.435.

(A)

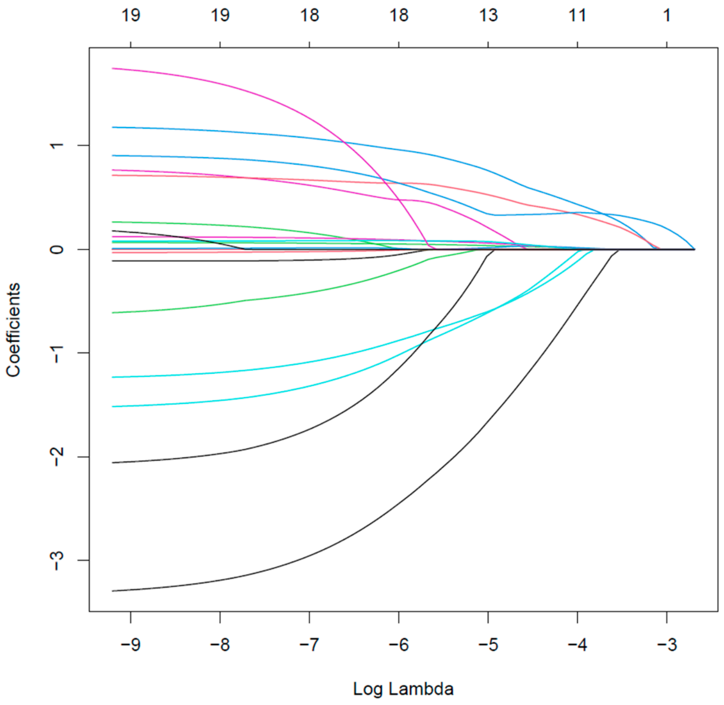

(B)

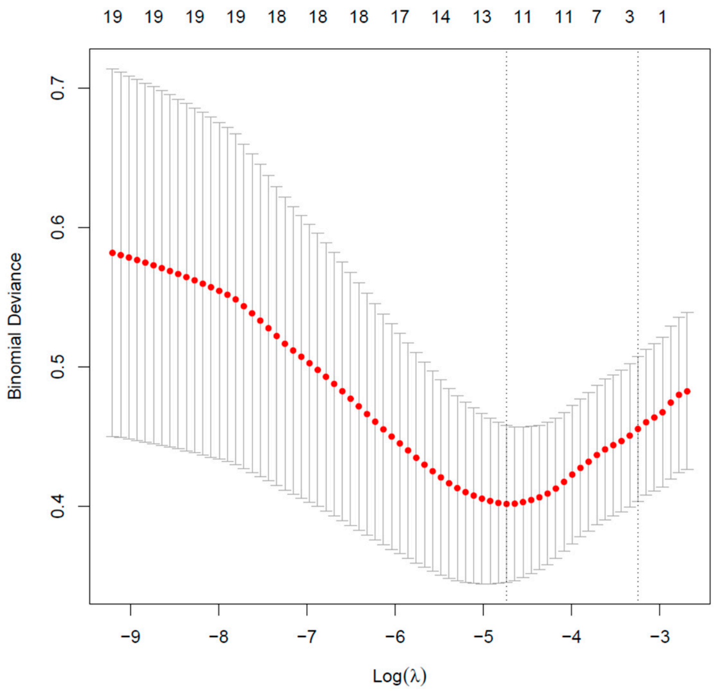
19 19 19 19 18 18 18 17 14 13 11 11 7 3 1
Binomial Deviance
0.4
0.5
0.6
0.7
−9 −8 −7 −6 −5 −4 −3
Log(λ)

(C)

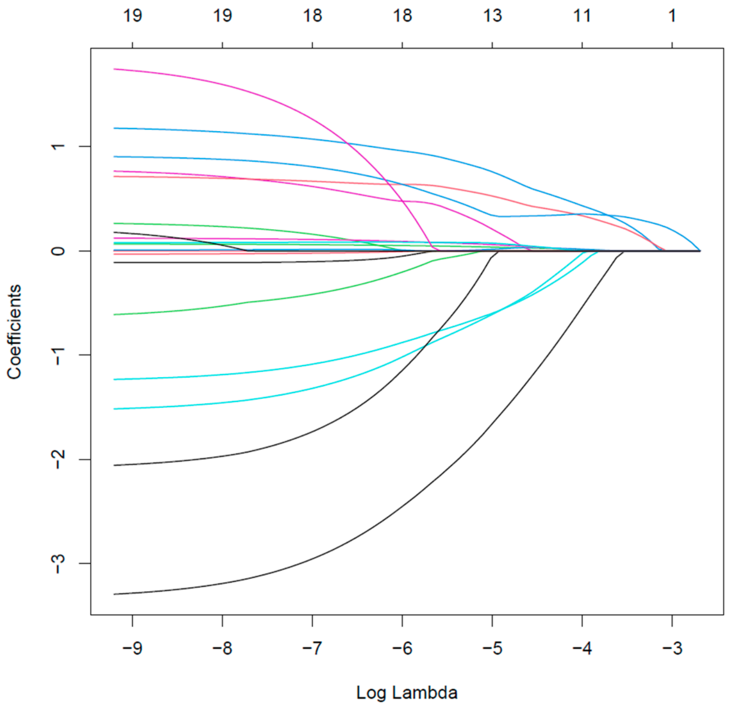
19 19 18 18 13 11 1
Coefficients
−3
−2
−1
0
1
−9 −8 −7 −6 −5 −4 −3
Log Lambda

(D)

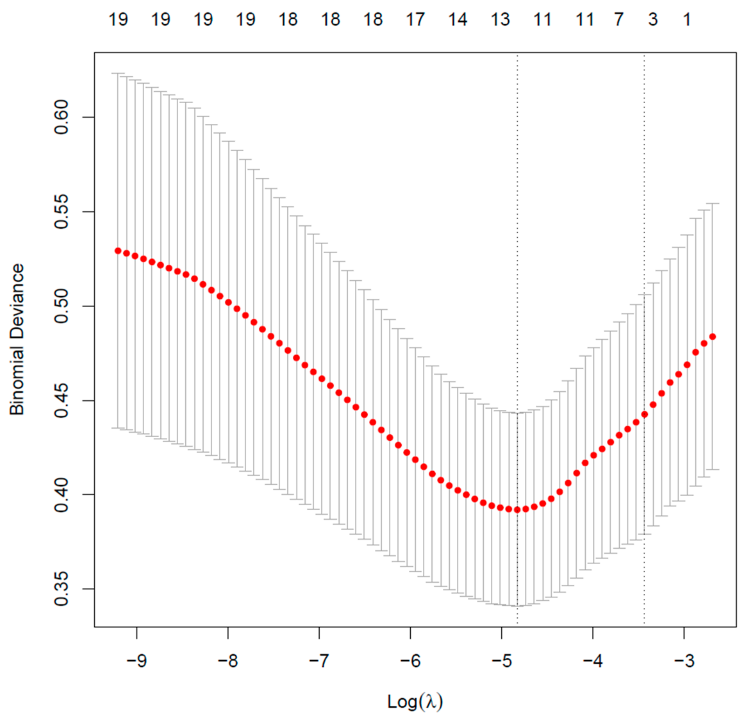
19 19 19 19 18 18 18 17 14 13 11 11 7 3 1
Binomial Deviance
0.60
0.55
0.50
0.45
0.40
0.35
−9 −8 −7 −6 −5 −4 −3
Log(λ)
